# Spatio-Temporal Differentiation Characteristics and Urbanization Factors of Urban Household Carbon Emissions in China

**DOI:** 10.3390/ijerph19084451

**Published:** 2022-04-07

**Authors:** Chen Li, Le Zhang, Qinyi Gu, Jia Guo, Yi Huang

**Affiliations:** 1 School of Management, Shanghai University of Engineering Science, Shanghai 201620, China; lichen@sues.edu.cn; 2 School of Marxism, Jiangnan University, Wuxi 214000, China; gqy2331@163.com (Q.G.); 6212403034@stu.jiangnan.edu.cn (J.G.); 3 School of Geographic Sciences, Nantong University, Nantong 226019, China; huangyi@ntu.edu.cn

**Keywords:** urban household carbon emissions, Exploratory Spatial Data Analysis (ESDA), Geographically Weighted Regression (GWR), China

## Abstract

This paper explores the spatial relationship between urbanization and urban household carbon emissions at the prefectural level and above cities in China and uses Exploratory Spatial Data Analysis (ESDA) and Geographically Weighted Regression (GWR) to reveal the extent of the impact of urbanization on urban household carbon emissions and the spatial and temporal variation characteristics. The results show that: Overall carbon emissions of urban households in cities of China showed a decreasing trend during the study period, but there were significant differences in the carbon emissions of urban households in the four major regions. In terms of the spatial and temporal characteristics of urban household carbon emissions, the urban “head effect” of urban household carbon emissions is obvious. The high-high clustering of urban household carbon emissions is characterized by a huge triangular spatial distribution of “Beijing-Tianjin-Hebei, Chengdu-Chongqing, and Shanghai”. The level of urbanization in Chinese cities at the prefecture level and above shows a spatial pattern of decreasing levels of urbanization in the east, middle, and west. The four subsystems of urbanization are positively correlated with urban household carbon emissions in the same direction. The urbanization factors have a contributory effect on some cities’ carbon emissions of urban households, but there are significant regional differences in the impact of urbanization factors on urban household carbon emissions in the eastern, central, and western regions of China, as they are at different stages of rapid urbanization development.

## 1. Introduction

Nobel Laureate Joseph Stiglitz once predicted that urbanization in China and high-tech development in the US would be the two major issues that would profoundly affect human development in the 21st century [1]. In fact, urbanization is significant and profound for China’s transformational development. On the one hand, rapid urbanization has energized China’s economic growth [2]; on the other hand, rapid urbanization has brought about problems such as carbon emissions [3,4,5]. Promoting low-carbon emission reduction is an important part of China’s efforts to achieve the United Nations Sustainable Development Goals (SDGs) and a major strategic decision by the Chinese government to achieve carbon peaking by 2030 and carbon neutrality by 2060. Studying the extent of the impact of urbanization on urban household carbon emissions and the characteristics of changes in space and time can provide a basis for urban household households to reduce emissions and is of great significance in promoting the reduction of urban household carbon emissions.

Various aspects of the relationship between urbanization and household carbon emissions have been discussed by academics at different spatial scales.

On a global scale, some scholars argue that there is a long-term equilibrium relationship between economic globalization, urbanization, and carbon dioxide emissions [6], while others argue that there is an inverted U-shaped relationship between urbanization and carbon emissions [7], and others argue that there is a clear correlation between population size, GDP per capita, carbon dioxide emission intensity, the share of fossil energy in total energy consumption and population structure and global carbon emissions [8]. However, the above scholars have not analyzed the extent to which urbanization has an impact on carbon emissions, whether positively or negatively. In response to this deficiency, some scholars have argued that urbanization may have an offsetting effect on carbon dioxide emissions, as it can increase environmental pressure through industrialization and reduce it through technological progress and economic restructuring [9], while the level of technological innovation, GDP per capita and information technology all contribute significantly to carbon efficiency [10]. In other words, it is possible that urbanization has a positive impact on carbon emissions. Is this finding appropriate for developing countries? Researchers have noted that the relationship between urbanization and carbon emissions is mainly positive for emerging developing countries [11]. There is an overall ‘inverted U’ relationship between urbanization and carbon emissions in the BRICS countries, with urbanization rate, population, GDP per capita, industrialization rate, energy intensity, and energy mix all being important influences on carbon emissions, but urbanization has the greatest impact on the rise of carbon emissions in the BRICS countries [12]. In the stage of rapid urbanization, it remains to be empirically analyzed how China’s urbanization affects carbon emissions, especially those of household households, whether positively or negatively.

At the national level in China, the urbanization rate, energy consumption per urban employed population, and carbon intensity of energy consumption show a positive effect [13]. For every percentage point increase in China’s urbanization rate, carbon emissions increase by 1.44 percentage points; for every percentage point increase in industrial value-added as a share of GDP, carbon emissions increase by 1.86 percentage points [14]. The relationship between urbanization and carbon emissions is an N-shaped curve in regions with low overall urbanization quality in China and an inverse N-shaped curve in regions with high and medium urbanization quality [15]. There is an inverted U-shaped relationship between per capita income and carbon emissions, with population size, industrial structure, and energy intensity all contributing in varying degrees to the growth of carbon emissions [16]. From a sub-regional perspective, the results of the empirical analysis of China’s sub-provinces show that population size is the most important influencing factor for urban carbon emissions [17]. In eastern China, there is an inverted U-shaped relationship between the square of GDP and CO_2_ emissions [18]. The relationship between the level of urbanization and carbon emissions in China’s three major urban agglomerations shows a ‘U-shaped’ curve that first falls and then rises [19]. There is a negative correlation between urbanization quality and carbon emissions in Yangtze River Delta cities, with carbon emissions decreasing by about 1.8% for every 1% increase in the urbanization quality index [20]. The effect of urbanization development on carbon emissions in Anhui Province is a multiplicative power function [21]. CO_2_ emissions from Beijing households show an increasing trend in urban areas but a slight decrease in rural areas, while higher population and urbanization lead to enhanced emissions in both urban and rural areas [22]. The above analysis helps us understand the relationship between urban household carbon emissions and urbanization in China during the rapid urbanization phase, but there are shortcomings, such as the lack of exploration of the spatial relationship between urbanization and urban household carbon emissions in China from a national sub-prefecture-level city perspective.

In terms of the relationship between carbon emissions and urbanization, scholars have tried to explain the impact of urbanization on carbon emissions. There are typically two types of views: first, urbanization leads to an increase in carbon emissions. Urbanization strengthens the rigid demand for energy consumption [23], the urbanization of land has a clear positive impact on carbon emissions [24], the continued rise in demand for transport travel, and the rise in household carbon emissions brought about by the increase in the living standards of residents [25]. Second, urbanization is conducive to reducing carbon emissions. Increasing urbanization rates have shown an overall dampening effect on the growth of carbon emissions [26], and urbanization development will be conducive to achieving carbon emission reductions in China [27]. Although urbanization development will increase carbon emissions in the short term, the long-term effect will contribute to carbon emission reduction [28], and urbanization will dampen this negative effect through the accumulation of human capital and the promotion of cleaner production technologies [29,30]. The two types of empirical analyses have different but valid points of view, and the reason for this is that urbanization factors have different degrees of influence on household carbon emissions at different stages of urbanization.

In addition to the above typical views, researchers have also analyzed the effects of urbanization-related factors such as the effects of residential consumption [31], population migration [32], urban lifestyle [33], income [34], the urban-rural structure of population [35] and household registration [36] on carbon emissions of urban households, providing a rich empirical experience for our study. However, there are some shortcomings in the above studies, mainly in two aspects: firstly, urbanization is a comprehensive variable, not only in terms of population urbanization, but also in terms of many aspects of the economy, lifestyle, and regional landscape. Therefore, it is necessary to strengthen the analysis of the impact of multiple factors of urbanization on the carbon emissions of households; secondly, the huge differences in natural conditions and levels of economic development in China have led to significant differences in the stages of urbanization development among the four major regions of China. Therefore, the study should not only examine the total impact of urbanization on urban household carbon emissions but also explore the differential impact on urban household carbon emissions in the four major regions.

The aim of this study is to reveal the extent of the impact of urbanization on urban household carbon emissions and the characteristics of the changes in space and time, in order to provide a basis for urban household carbon reduction. The study is divided into seven parts. The first part is the introduction. The second part is the research methodology and data sources. The third part is a spatial and temporal analysis of the characteristics of carbon emissions of urban households. The fourth part is a comprehensive measure of China’s urbanization level. The fifth part is a spatial analysis of the urbanization factors of carbon emissions from urban households. The sixth part is the discussion and conclusions. The seventh part is the suggestions.

## 2. Methods and Data

### 2.1. Methods

#### 2.1.1. Entropy Method (EM)

This study uses the entropy method to comprehensively evaluate the level of urbanization development of 286 prefecture-level and above cities in China. Entropy is a physical concept that originated from thermodynamics and was later introduced into information theory by C∙E∙Shannon and is now widely used in social and economic research fields. In information theory, entropy is a measure of the degree of disorder in a system, while information is a measure of the degree of order in a system, and the two are equal in absolute value and opposite in sign; if the degree of variation in the value of an indicator is greater, the lower the entropy, the greater the amount of information provided by the indicator, and the greater its weight should be; conversely, the less variation in the value of an indicator, the greater the entropy, the smaller the amount of information provided by the indicator, and the smaller its weight [37]. Therefore, the entropy method can profoundly reflect the utility value of the entropy value of indicator information, and the indicator weight value given has higher credibility than the hierarchical analysis method and the expert experience assessment method, which is suitable for the comprehensive evaluation of multiple indicators, and its main steps are:

Step one: construct the original indicator data matrix: assume there are *m* programs to be evaluated and *n* evaluation indicators, forming the original indicator data matrix:

(1) $$X={\{x_{i}{}_{j}\}}_{m\times n}(0\leq i\leq m,0\leq j\leq n)$$

where *x_ij_* is the indicator value of the *j*th indicator of the *i*th program to be evaluated.

Step two: data standardization: as the scale, order of magnitude and positive and negative orientation of the indicators are different, the initial data should be standardized.

(2) $$x_{ij}^{\prime }=\left\{ \begin{array}{l} \frac{x_{ij}-x_{ij\min}}{x_{ij\max}-x_{ij\min}}\\ \frac{x_{ij\max}-x_{ij}}{x_{ij\max}-x_{ij\min}} \end{array}\right.$$

Define the normalization matrix:

(3) $$y_{ij}=\frac{x_{ij}^{\prime }}{\sum x_{ij}^{\prime }},0\leq y_{ij}\leq 1$$

Step three: let *k* = 1/ln *m*, calculate the entropy value of the evaluation indicator:

(4) $$e_{j}=-k\sum (y_{ij}\times \ln y_{ij})=(\frac{1}{\ln m})\sum \left(y_{ij}\times \ln y_{ij}\right)$$

Step four: calculate the coefficient of variability of the evaluation indicators and define the weights of the evaluation indicators:

(5) $$g_{j}=1-e_{j}$$

(6) $$w_{j}=g_{j}/\sum g_{j}$$

Step five: calculate the evaluation value of the sample: the product of the weight *w_j_* of the *j*th indicator and the proximity *x′_ij_* of the *j*th evaluation indicator of the *i*th sample in the standardized matrix is used as the evaluation value *f_ij_* of *x_ij_*, and the evaluation value *f_i_* of the *i*th sample.

(7) $$f_{ij}=w_{j}\times x_{ij}^{\prime }$$

(8) $$f_{i}=\sum f_{ij}$$

#### 2.1.2. Exploratory Spatial Data Analysis (ESDA)

The technique of spatial autocorrelation originates from Tober’s idea of the first law of geography, which states that everything is spatially correlated and that there is a decay of distance between things, with the closer things are to each other, the more likely they are to be spatially correlated. Spatial autocorrelation includes global spatial autocorrelation and local spatial autocorrelation. The Moran’s *I* statistic is generally used to measure global spatial autocorrelation, and the formula for calculating the global Moran’s *I* statistic is [38]:

(9) $$I=\frac{\sum_{i=1}^{n}\sum_{j=1}^{n}w_{ij}(x_{i}-x^{\prime })(x_{j}-x^{\prime })}{S^{2}\sum_{i=1}^{n}\sum_{j=1}^{n}w_{ij}}$$

In the equation, the index *I* is the global Moran’s statistic, *n* is the total number of samples, *x_i_*(*x_j_*) is the household carbon emission indicator for the *i_j_*th city and is the mean value of the household carbon emission indicator for each city. *w_ij_* is a binary variable reflecting the proximity of two cities, 1 for proximity and 0 for non-proximity, *i* ranges from [−1, 1], with values greater than 0 indicating positive correlation, less than 0 indicating negative correlation and equal to 0 indicating no correlation. The global Moran index can be determined by constructing a statistic *Z* that follows a normal distribution and adopting a two-tailed test to determine whether there is a significant spatial correlation among the *n* regions. *I* denotes Moran’s *I* statistic; *E*(*I*) denotes the expectation of Moran’s *I*; *VAR*(*I*) denotes the variance of Moran’s *I*. The variance of the urban household carbon emissions indicator is:

(10) $$S^{2}=\frac{1}{n}{\sum_{i=1}^{n}\left(x_{i}-x^{\prime }\right)}^{2}$$

The *Z*-statistic is calculated as:

(11) $$Z=\frac{I-E(I)}{\sqrt{VAR(I)}}$$

Local spatial autocorrelation analysis is used to measure the spatial clustering characteristics of local subsystems and to explore the spatial variation and significance of household carbon emission indicators between a city and other neighboring cities in China. The local Moran’s *I* statistic is generally used to measure the local spatial dispersion of urban household carbon emissions through LISA clustering maps. The local Moran’s *I* statistic is defined as follows.

(12) $$I_{i}=z_{i}\sum_{j=1}^{n}w_{ij}z_{j}$$

where *z_i_* and *z_j_* are the deviations of the household carbon emission indicators from the mean for the *i*th and *j*th cities, *z_i_* = (*x_i_’*) and *z_j_* = (*x_j_’*). *w_ij_* is a normalized spatial weight matrix with all diagonal elements being 0. At a given significance level, *I_i_* > 0 indicates a positive correlation with similar values clustering in neighboring cities; *I_i_* < 0 indicates a negative correlation with no similar values clustering in neighboring cities clustering. Combined with the LISA significance level, the LISA clustering map can identify the “cold spots” and “hot spots” of urban household carbon emissions in local space, revealing the spatial heterogeneity of the development of urban household carbon emissions.

#### 2.1.3. Geographically Weighted Regression (GWR)

Due to the complexity and variability of spatial data, GWR models assume heterogeneity of correlates between regions, making local spatial analysis more realistic to address. Geographically weighted regression (GWR), a local form of linear regression, is used to model spatially varying relationships. spatial measures of this method have been widely used since the introduction of geographically weighted regression by Fotheringham et al. in 1996. GWR model is an extension of the ordinary regression model and is an estimate of local spatial relationships. Therefore, a global estimation of the data must be performed before applying the model, followed by an analysis of the local spatial relationships.

The regression coefficients of the GWR independent variables vary with spatial location and the regression coefficients of the GWR model are estimated locally by weighted least squares with weights determined according to the idea of Tobler’s first law. The general form of the GWR model is [39]

(13) $$y_{i}=\sum_{j=1}^{p}\beta_{j}(u_{i,}v_{i})x_{ij}+\varepsilon_{i},i=1,2,\cdots ,n;j=1,2,\cdots ,n$$

In the formula, *x_i_*_1_, *x_i_*_2_,…, *x_ip_* are the observed values of the independent variables *x*_1_, *x*_2_,…, *x_p_* at the spatial location (*u_i_*, *v_i_*) out, *i* is the coefficient to be estimated, *i* is the error term, and the coefficient *i* to be estimated is calculated by the weighted least squares method.

### 2.2. Data

The carbon emission data of 286 cities at the prefecture-level and above in China were collected from the China City Greenhouse Gases Emission Dataset (2005) and China City Greenhouse Gases Emission Dataset (2015) published by the China Environment Publishing Group and authored by the China Urban Greenhouse Gas Working Group [40,41]. Data on the urbanization factor variables of the urban household of carbon emissions were collected from China Urban Statistical Yearbook 2016 [42] and China Urban Construction Statistical Yearbook 2015 [43].

## 3. Spatial and Temporal Variations in Carbon Emissions of Urban Households

### 3.1. Characteristics of the Time-Series Evolution of Urban Household Carbon Emissions

This section analyses the spatial and temporal patterns of urban household carbon emissions in China’s cities from three perspectives: overall, by prefecture-level cities, and by four regions, revealing the “east, middle, and west” gradient pattern of urban household carbon emissions in China.

Overall, urban household carbon emissions in 286 cities at the prefecture-level and above in China showed a trend of shrinking from 2005 to 2015. Both in terms of total and mean data, urban household carbon emissions have been declining over the study period. Both the total and average urban household carbon emissions decreased by 22% over the study period, meaning that urban household carbon emissions in 2005 were 1.28 times lower than those in 2015.

**Figure 1:**
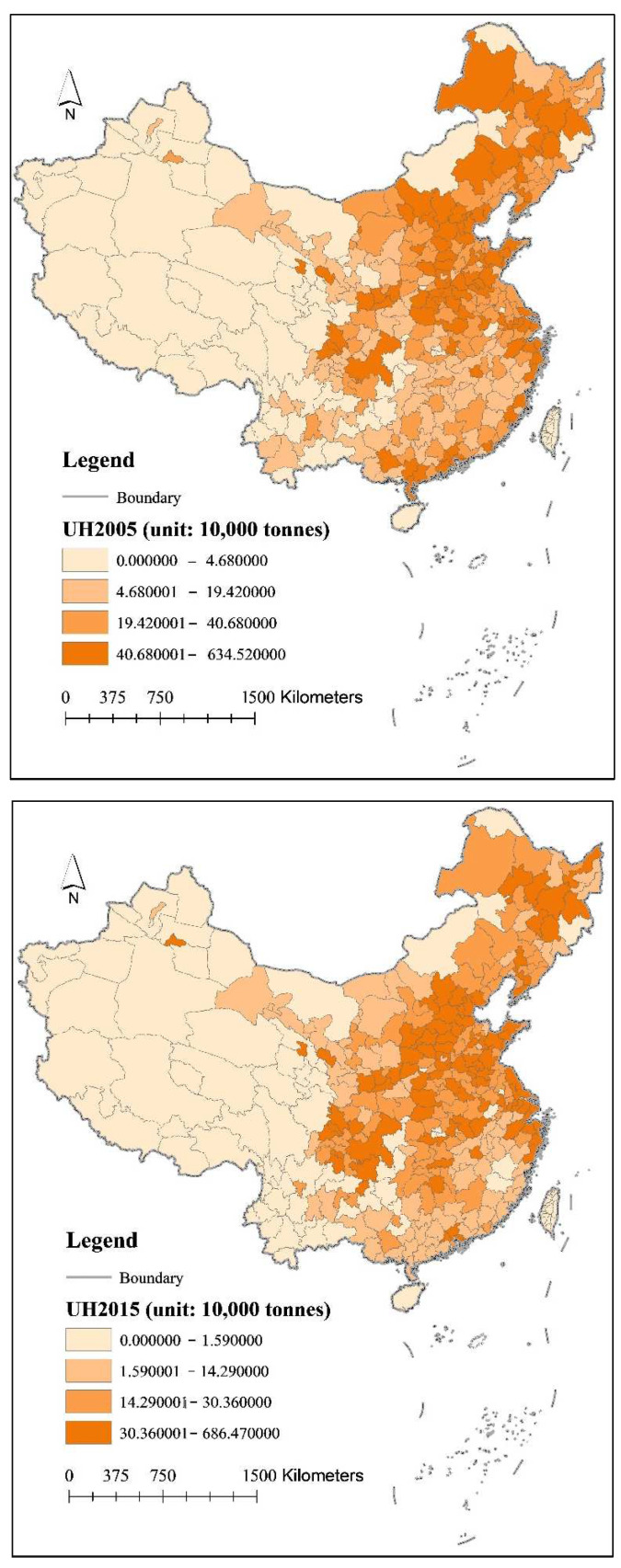
Spatial and Temporal Distribution of Carbon Emissions from Urban Households in China (2005–2015).

At the prefecture-level city level, using the quartile method to classify urban household carbon emissions, it was found that urban household carbon emissions were generally higher in eastern China than in central and western China in 2005, and this spatial pattern did not change significantly in 2015 (Figure 1). However, from 2005 to 2015, important cities in western China, such as Chongqing and Chengdu, showed a significant increase in household carbon emissions. In terms of the north-south spatial pattern, China’s livable zones such as the Northeast Plain, the North China Plain, the Yangtze River Economic Belt, and the Sichuan Basin have consistently maintained high levels of urban household carbon emissions. It is very surprising that these livable zones are among the key areas of China’s urbanization development and are the core areas of China’s population concentration. Of course, to what extent do urbanization factors influence the carbon emissions of urban households, and what are the main influencing factors that manifest themselves? We need to further verify this using quantitative methods.

Decomposition and analysis of urban household carbon emissions in the eastern, western, northeastern, and central regions of China (Table 1).

Firstly, looking at the eastern region, whether in terms of the total carbon emissions of households in the four major regions or the average carbon emissions of households in each region, China’s urban household carbon emissions have a spatial pattern of being higher in the eastern region than in the central and western regions. The eastern coastal region is the most economically developed region in China and is one of the regions with the highest concentration of urban population and the highest level of urbanization, which undoubtedly brings about an increase in carbon emissions from urban households.

Secondly, looking at the western region, although overall the population density and urbanization level of the western region is not as high as that of China’s eastern region, based on the law of urbanization, since the Chinese government implemented the Western Development Strategy in 1999, the provincial capitals and municipalities directly under the Central Government in the western region have been developing rapidly, and their infrastructure development, urbanization level, and urban built-up area density have been significantly enhanced. This is the reason why Chongqing and Chengdu have seen their carbon emissions rise in the face of the decline in overall urban household emissions in China.

Thirdly, looking at the northeast region, which, as China’s old industrial base, has made a remarkable contribution to the industrialization of the country and the establishment of a complete industrial system since the founding of New China. However, with the decline of the old industrial bases, the outflow of population from the northeast has become a serious problem, and “shrinking cities” are a typical feature of the three northeast provinces. In terms of both total and average urban household carbon emissions, the northeast has seen a significant reduction in urban household carbon emissions.

Fourthly, looking at the central region, compared to the urban household carbon emissions in the northeast, east, and west, the urban household carbon emissions in the central region seem to be lukewarm. No significant reduction or change in either the total or average urban household carbon emissions is observed.

In summary, urban household carbon emissions in the four major regions are to some extent related to urbanization factors. The developed eastern coastal regions of China are the engines of China’s economic development, attracting large numbers of people there and accelerating urbanization, while the northeastern regions are the exact opposite, with cities decaying due to economic decline and population outflow. The core cities in the central and western regions, as engines driving regional development, are accelerating development and are attracting people to urbanize locally. The direct result is that carbon emissions from core urban households in some of the central and western regions are significantly higher, not significantly lower.

### 3.2. Spatially Divergent Characteristics of Urban Household Carbon Emissions

This section reveals the spatially heterogeneous characteristics of carbon emissions of urban households in terms of two dimensions: rank-size and local spatial autocorrelation, respectively.

**Figure 2:**
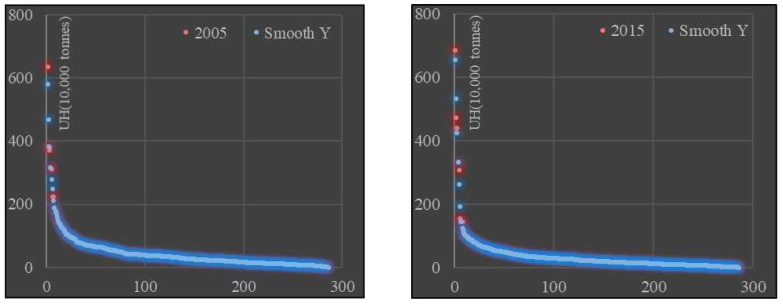
Urban household carbon emissions rank-size distribution.

The rank-size analysis shows that there is a clear “head effect” on carbon emissions for urban households, with significant differences within the four regional groups. In terms of overall differences, the carbon emissions of households in 286 cities at prefecture level and above were ranked and smoothed, and it was found that there was a clear “head effect” in the carbon emissions of households in 286 cities, with the carbon emissions of households in the top 10% of cities significantly higher than those in the bottom 90% of cities (Figure 2). In terms of regional differences, between 2005 and 2015, the number of cities with urban households emitting more than 1 million tons of carbon fell from 23 to 12, and the Theil index of urban household carbon emissions expanded from 0.529 to 0.695, with the intra-group difference expanding from 0.485 to 0.685 and the inter-group difference narrowing from 0.045 to 0.010, revealing significant differences in urban household carbon emissions within China’s four major regions. Urban household carbon emissions differ significantly.

Local spatial autocorrelation analysis shows that in 2005, there were 22 cities with an HH cluster of urban household carbon emissions in China, including two in the northeast, 16 in the east, two in the central region, and two in the west; four cities with an HL cluster, including one in the central region and three in the west; and one city with an LH cluster, a city in Inner Mongolia in the west. However, China’s urban Household carbon emissions in 2015 were characterized by an HH cluster, with 13 cities in an HH cluster, including seven in the eastern region, two in the central region, and four in the western region (Table 2).

Changes in the type of carbon emission cluster of urban households in China from 2005 to 2015: first, Beijing, Tianjin, Tangshan, Baoding, Shijiazhuang, Handan, and Shanghai in the eastern region continued to maintain the characteristics of an HH cluster; second, Chengdu and Chongqing in the western region changed from the characteristics of an HL cluster in 2005 to an HH cluster in 2015; third, Xi’an and Deyang in the western region evolved from an insignificant spatial cluster in 2005 to an HH cluster.

**Figure 3:**
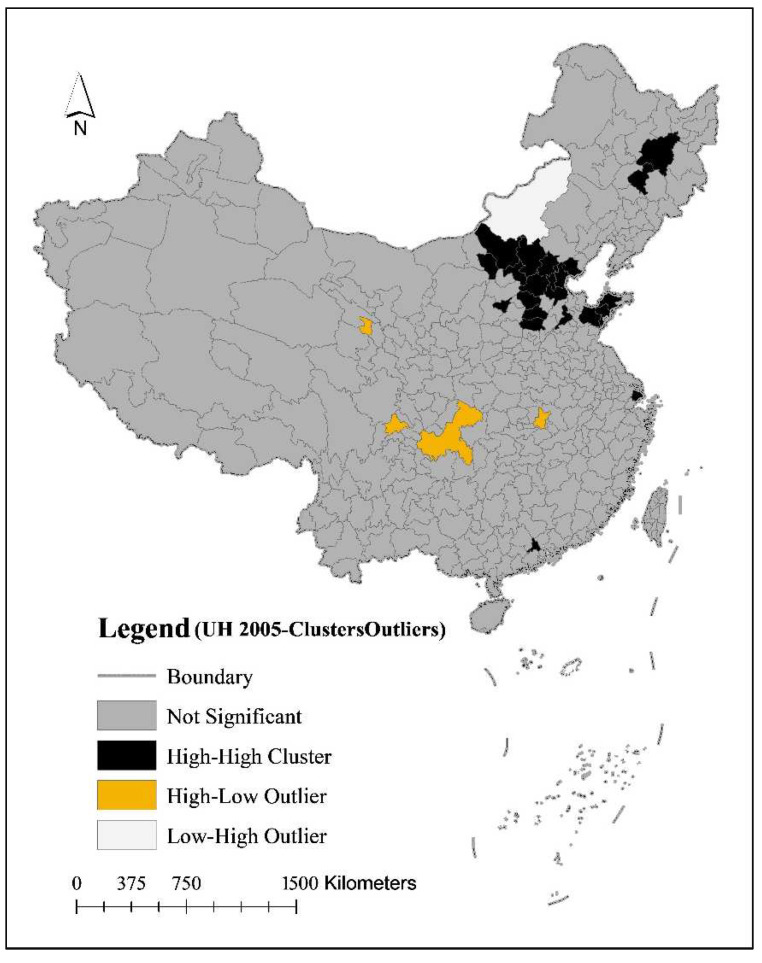

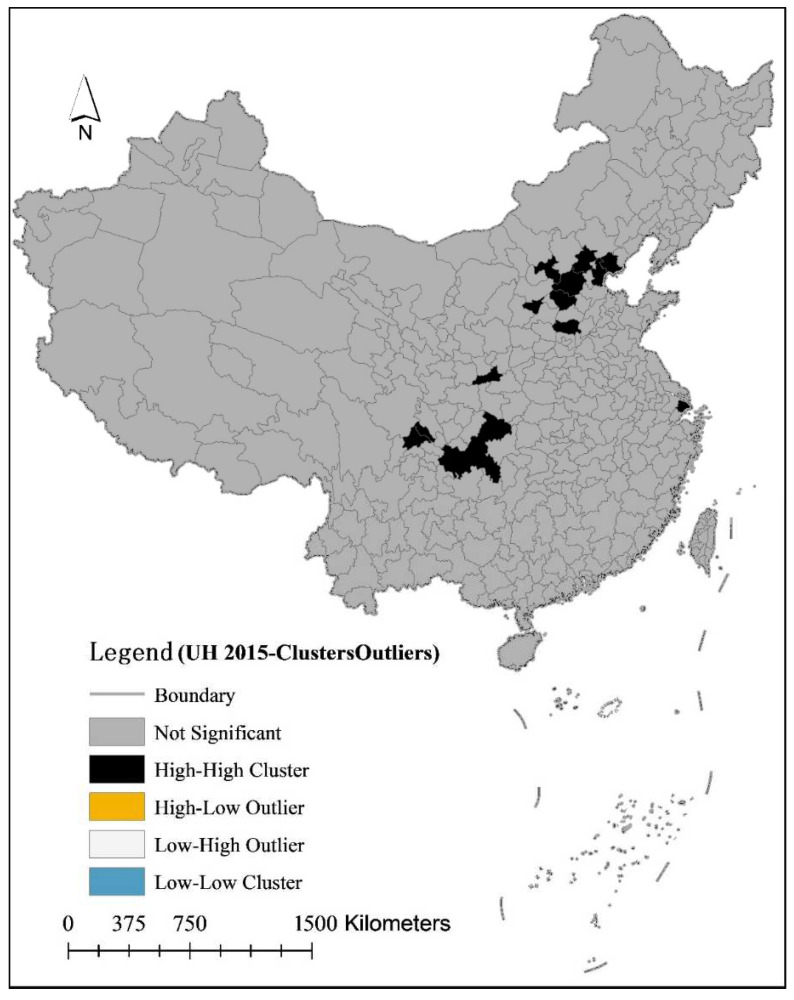
Local spatial autocorrelation of carbon emissions from urban households in China.

Analysis of the spatial agglomeration characteristics of carbon emissions from urban households (Figure 3).

Firstly, the spatial agglomeration of urban household carbon emissions is an important region for China’s economic development and livability. The 2005 HH agglomeration and HL agglomeration are distributed in the Northeast Plain, the North China Plain, the Sichuan Basin, and the Yangtze River Delta region; in 2015, the spatial agglomeration of urban household carbon emissions in the northeast region is no longer significant, and the HH agglomeration of urban household carbon emissions shows a huge triangle of “Beijing-Tianjin-Hebei, Chengdu-Chongqing, and Shanghai”.

Secondly, the spatial clustering of urban household carbon emissions is characterized by provincial capitals and municipalities directly under the central government. Such as 13 cities, including Harbin, Changchun, Beijing, Tianjin, Shijiazhuang, Jinan, Shanghai, Taiyuan, Hohhot, Xining, Chengdu, Chongqing, and Wuhan, were the provincial capitals and municipalities directly under the central government in 2005, accounting for 59.09% of the total, municipalities directly under the Central Government were eight cities including Beijing, Tianjin, Shijiazhuang, Shanghai, Taiyuan, Xi’an, Chengdu, and Chongqing, accounting for 61.54%.

## 4. Construction of Urbanization Level Indicators and Comprehensive Measurement

### 4.1. Urbanization Level Indicator Construction

The first step in measuring the level of urbanization and its comprehensive measurement is to clarify the definition of urbanization. The process of socio-economic development from an agricultural society to an industrial society is characterized by a gradual increase in the proportion of non-agricultural activities, and the population inevitably shifts from rural areas, where agricultural activities are the mainstay, to urban areas, where non-agricultural activities are the mainstay, and this is the basic meaning of urbanization [44,45,46,47,48,49,50]. Many different measurement methods have been proposed by academics for measuring the level of urbanization, and in summary, they are mainly divided into two types: the single indicator method and the composite indicator method [51,52,53,54,55,56]. The single-indicator method mainly calculates the proportion of the urban population, the proportion of the non-agricultural population, and the proportion of urban land [37], but it has two shortcomings: firstly, it only reflects a certain aspect of urbanization and lacks a comprehensive analysis; secondly, due to the adjustment of administrative divisions, the statistical calibers of urban population is missing [57,58,59,60,61,62,63], which makes it difficult to accurately reflect the actual situation of urbanization. Compared with the single-indicator method, the composite indicator method can reflect the level of regional urbanization development in a more comprehensive and complete way and is increasingly accepted by the academic community [64,65,66,67,68]. On the basis of the existing indicator systems [69,70,71,72,73,74], the study constructs a comprehensive evaluation index system of the urbanization level of cities from four aspects: population urbanization, economic urbanization, lifestyle urbanization, and geographical landscape urbanization (Table 3).

### 4.2. Comprehensive Measure of Urbanization Level

The entropy method was used to measure China’s comprehensive urbanization score to reveal the urbanization characteristics of Chinese cities at the prefecture-level and above, and the results showed that:

Firstly, the urbanization level of cities at the prefecture-level and above in China shows a spatial pattern of decreasing in the order of “east, central and west”. The urbanization level of the eastern region is higher than that of the central region, while the urbanization level of the central region is higher than that of the western region, and the urbanization level of the northeastern region is between that of the central region and the western region. The mean urbanization composite scores of the eastern, central, western, and northeastern regions are 0.3248, 0.2928, 0.2682, and 0.2793, respectively.

Secondly, by dividing the ranking of the four regions into the top 25%, middle 50%, and bottom 25% of the urbanization composite score, it is found that the number of cities in the top 25% of the urbanization composite score in the eastern region leading position, while the number of cities in the bottom 25% of the urbanization composite score in the eastern region is relatively small. The number of cities in the top 25% of the combined urbanization score in the eastern region is 34, 16 in the central region, 16 in the western region, and six in the northeastern region. The number of cities in the bottom 25% of urbanization scores in the eastern region was 10, 16 in the central region, 40 in the western region, and six in the north-eastern region (Table 4).

**Figure 4:**
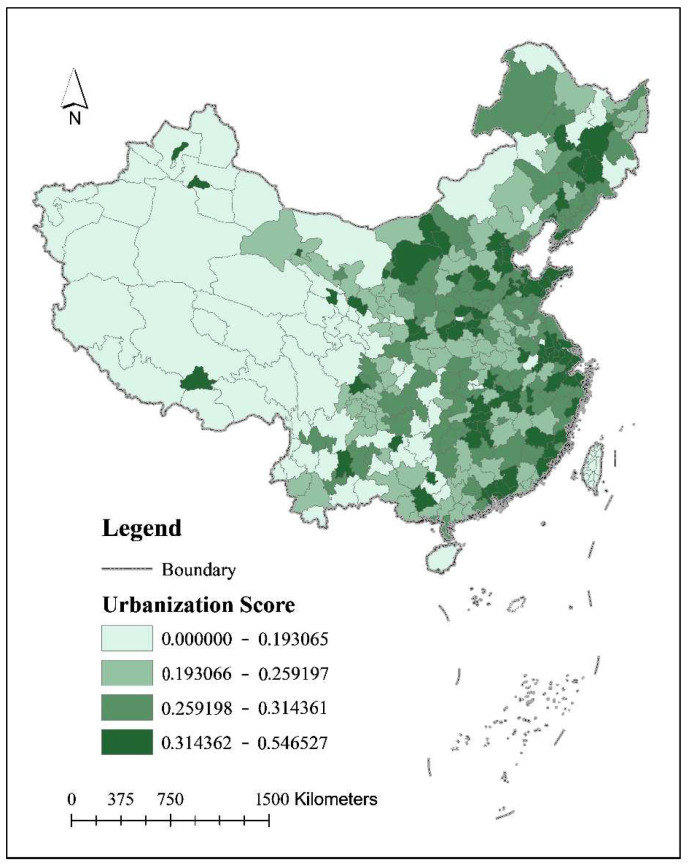
China’s urbanization level composite score.

Thirdly, China’s large urban agglomerations have a high overall urbanization score, showing a “polycentric” trend towards the central cities of urban agglomerations, which is clearly different from the “head effect” of carbon emissions from urban households, reflecting the leading role of China’s large urban agglomerations in promoting urbanization (Figure 4). This reflects the leading role of China’s large urban agglomerations in promoting high-quality development. In terms of spatial distribution, the urbanization levels of national city clusters such as the Ha-Chang City Cluster, the Beijing-Tianjin-Hebei City Cluster, the Shandong Peninsula City Cluster, the Yangtze River Delta City Cluster, the Yangtze River Midstream City Cluster, the Central Plains City Cluster, the Guanzhong Plain City Cluster, the Southeast Fujian City Cluster, and the Guangdong-Hong Kong-Macao Greater Bay Area are high (Figure 4). Urban clusters are growing into an important strategic pivot point for China’s urbanization transformation and development in the new era. With a spatial pattern of less land, more employment, strong factor gathering capacity and reasonable population distribution, urban agglomerations have grown into engines of China’s economic development with strong driving and radiating effects within and among urban agglomerations. China’s national strategy of urban agglomerations is also in line with the law of urbanization and has formulated the outline of national economic and social development planning. The outline of the 11th Five-Year Plan clearly states that “urban agglomerations should be taken as the main form to promote urbanization, and gradually form a spatial pattern of urbanization that is efficient, coordinated and sustainable, with the coastal and Beijing-Guangzhou-Beijing-Harbin lines as the vertical axis, the Yangtze River and Longhai lines as the horizontal axis, a number of urban agglomerations as the main body, other cities and small towns distributed in a dotted pattern, and permanent arable land and ecological functional areas spaced apart”.

The secondary indicator scores are further analyzed in terms of demographic urbanization, economic urbanization, lifestyle urbanization, and geographical landscape urbanization (Table 5).

In terms of the ranking of population urbanization scores, the gradient of TOP25% and Middle50% is still evident, with the quality of population urbanization in eastern China being higher than that in central China, central China being higher than that in western China, and western China being slightly higher than that in northeastern China. In the past 20 years, China has been in a stage of rapid urbanization development, with an urbanization rate of 49.68% in the 6th Chinese census in 2010 and 63.89% by 2020, implying that China’s population urbanization is becoming more and more mature, with the trend of metropolitanization and concentration of urban agglomerations intensifying.

In terms of economic urbanization score ranking, the gradient of TOP25% and Middle50% is more obvious. Forty of the TOP25% cities are in the eastern region, 14 in the central region, 12 in the western region, and six in the northeastern region indicating that the quality of economic urbanization is closely related to the degree of population and economic development, and the developed eastern coastal region of China has become an important node region for carrying economy, population, science and technology, and so on, in important nodal regions.

In terms of lifestyle urbanization score ranking, TOP25% of cities in the central-eastern region, central region, western region, and northeastern region accounted for 12.24%, 4.55%, 5.94%, and 2.4% respectively, while Bottom25% of cities in the central-eastern region, central region, western region, and northeastern region accounted for 3.50%, 5.24%, 12.24%, and 4.20% respectively. It is still the eastern region that scores the highest.

In terms of geographical landscape urbanization scores, the TOP25%, central, western, and northeastern regions accounted for 11.54%, 7.69%, 4.55%, and 1.40% respectively, while the Bottom25%, central, western, and northeastern regions accounted for 3.15%, 5.94%, 11.54%, and 4.55% respectively. The eastern region scored the highest.

In summary, China’s urbanization and its subsystem scores show a gradient of decreasing urbanization quality in the east, middle and west, which is somewhat similar to the spatial distribution of urban household carbon emissions, implying that the forces of urbanization may have some influence on household carbon emissions. During the period of rapid urbanization, the government promoted the development of the economy through investment and the transfer of the population away from agriculture, bringing about an increase in the size of the urban population and driving up the carbon emissions of urban households. In the later stages of urbanization, as technology advances and economic efficiency improves, the more efficient the exploitation of urban resources is at this stage, the power of urbanization will bring about transformational economic development and technological progress, which will promote the intensive use of household carbon emissions, which will, in turn, be conducive to the reduction of urban household carbon emissions.

## 5. Analysis of Urbanization Factors of Carbon Emissions of Urban Households

### 5.1. Correlation Analysis of Urban Household Carbon Emissions with Urbanization Factors

**Figure 5:**
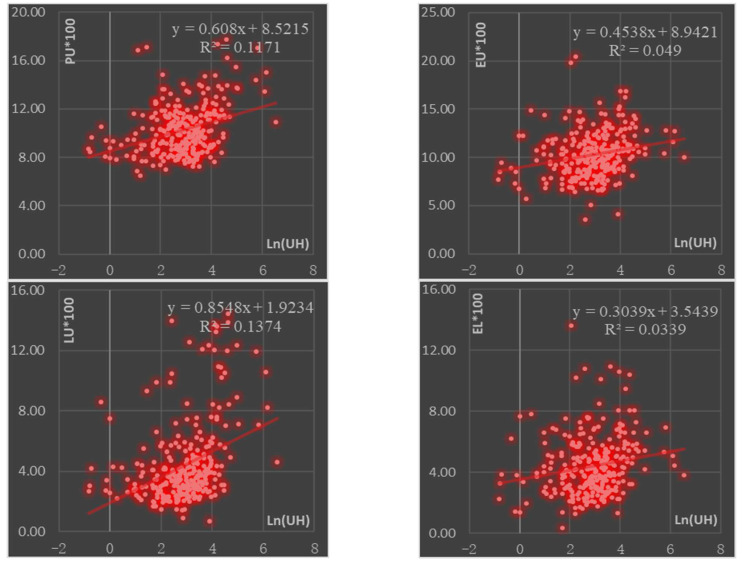
Scatterplot of urban household carbon emissions and urbanization indicators in China.

The spatial distribution of carbon emissions in China’s 286 cities at the prefecture-level and above shows that cities with high urban household carbon emissions also have higher overall urbanization scores. We plotted the scatter plots of population urbanization and urban household carbon emissions, economic urbanization and urban household carbon emissions, lifestyle urbanization and urban household carbon emissions, and landscape urbanization and urban household carbon emissions (Figure 5), and the results show that the four subsystems of urbanization are consistent with urban household carbon emissions in terms of direction.

In terms of correlation strength, lifestyle urbanization has the highest correlation strength with urban household carbon emissions, with a correlation coefficient of 0.37, while the lowest correlation strength is found for landscape urbanization, with a correlation coefficient of 0.18, reflecting the differences in the correlation between different levels of urbanization forces and urban household carbon emissions. At the significance level, demographic urbanization, economic urbanization, lifestyle urbanization, and landscape urbanization all pass the test, indicating that the correlation analysis is significant.

In the correlation analysis of the indicators of population urbanization, the correlation coefficients of the indicators of urban population to the total population, population size in urban areas, population density in urban areas, and the proportion of workers in the tertiary sector, except for the indicator of the proportion of workers in the secondary sector, were all positive, indicating that most of the indicators of population urbanization and carbon emissions from urban households are in the same direction. The correlation coefficient between urban population size and urban household carbon emissions is 0.746, which is a strong correlation between the two.

In the correlation analysis of economic urbanization indicators, the correlation coefficients of GDP per capita, GDP growth rate and the share of tertiary industry in GDP are positive, except for the two indicators of the share of secondary industry in GDP and the per capita gross industrial output value, indicating that most of the indicators of economic urbanization are homogeneous with the carbon emissions of urban households.

In the correlation analysis of lifestyle urbanization indicators, the correlation coefficients for the number of university students per 10,000 people, the number of urban employees per 10,000 people at the end of the period, and the retail sales of social consumer goods per capita are positive, indicating that their growth is in the same direction as the carbon emissions of urban households. The correlation coefficient for postal and telecommunications business per capita is negative but does not pass the significance test.

In the correlation analysis of the indicators of urbanization in the geographical landscape, except for the indicator of urban road area per capita, the correlation coefficients of the indicators of urban construction land as a proportion of urban area, built-up area per capita, fixed asset investment per capita and green coverage of built-up areas were all positive, indicating that most of the indicators of urbanization in the geographical landscape were homogeneous with the carbon emissions of urban households. However, most of the correlation coefficients of the indicators of urbanization in geographical landscapes failed the significance test.

Correlation analysis can indicate the interrelationship between variables, reflect the strength of correlation and the direction of effect between variables, and is an important reference value for exploring the causal relationship between variables (Table 6). The following further uses spatial analysis techniques to explore the spatial differences in the effects of urbanization factors on carbon emissions of urban households in China.

### 5.2. Geographically Weighted Regression Analysis

A geographically weighted regression model was developed with urban household carbon emissions (UH) as the dependent variable and the level of population urbanization, economic urbanization, lifestyle urbanization, and territorial landscape urbanization as the independent variables. Based on the spatial data attributes and distribution characteristics of the geographically weighted regression analysis, the AICc criterion was chosen to determine the bandwidth of the GWR weights. AICc not only measures the difference between the observed and fitted values but also provides a measure of the model complexity.

**Figure 6:**
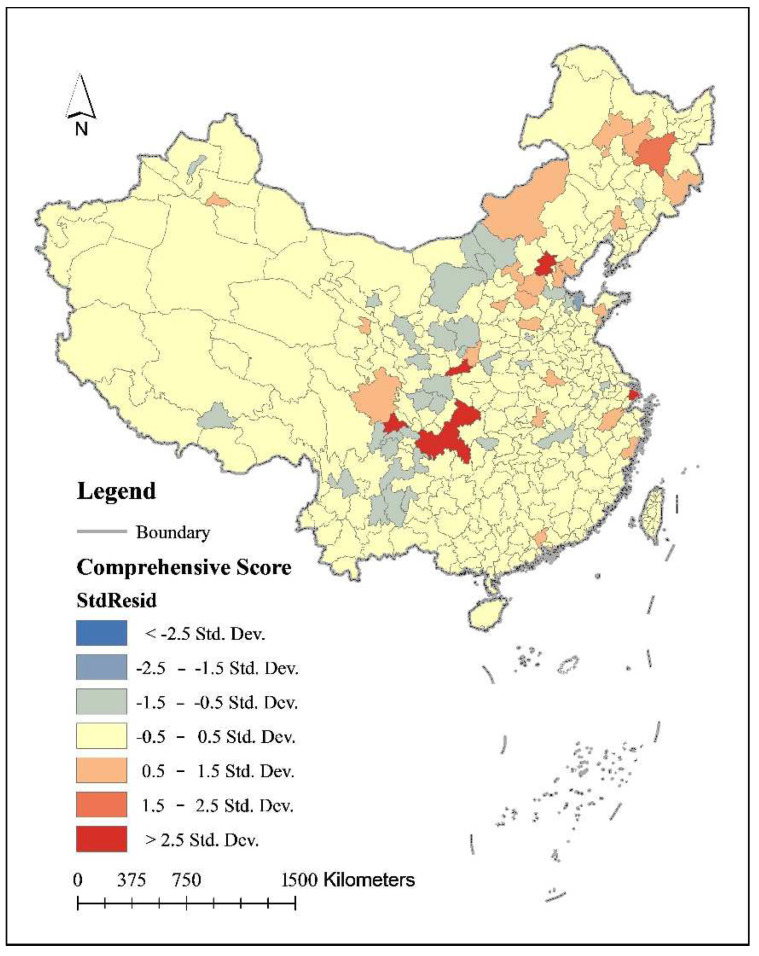
Geographically weighted regression analysis of urbanization factors on carbon emissions of urban households in China.

The geographically weighted regression model was used to measure Bandwidth, Residual Squares, Siga, AICc, R^2^, and R^2^ Adjusted values, which are 478,493.34, 88,012,839, 51.64, 3992.14, 0.28, and 0.20 respectively, where R^2^ Adjusted reveals in general terms the extent to which the factors of level of demographic urbanization, level of economic urbanization, level of lifestyle urbanization and level of territorial landscape urbanization explain the carbon emissions of household households, in this case, 20%, that’s the urbanization variables in this study explain 20% of the factors of carbon emissions of household households overall. The study used GIS software to symbolize the residuals of the GWR measurement processing (Figure 6).

First, the residual analysis, StdResid, reflects the difference between the observed and fitted values, the smaller the difference, the better the fit, and the distribution of high and low residuals reflects the spatial differences in carbon emissions of urban households fitted in the GWR model. In general, most cities in China have a good fit and the differences between the observed and fitted values are small.

Second, the local R^2^ analysis. The change in local R^2^ reflects the degree to which each indicator in the GWR model explains the carbon emissions of urban households. The higher the local R^2^, the higher the degree of explanation. the results of the GWR regression analysis show that the local R^2^ of northern Chinese cities is generally higher than that of southern cities, the local R2 of northwest China and northeast China is the highest, the local R^2^ of south China and southwest China is lower, the lowest local R^2^ is in Hainan Province, and the local R^2^ of intermediate zones such as east China and north China is in the middle, and its degree of explanation is also in the middle.

Third, the analysis of variable parameters. From the variable parameters of population urbanization, eastern China, which extends westwards all the way along the Yangtze River to the Yunnan-Guizhou Plateau, is the region where the population urbanization factor has the greatest impact on the carbon emissions of urban households. The Yangtze River is one of the sources of Chinese civilization and one of the most livable economic zones in China. Population urbanization and high-quality development along the golden waterfront of the Yangtze River Economic Zone are of great significance in promoting energy conservation, emission reduction, and a low-carbon economy, and the population agglomeration in livable areas has a driving effect on the growth of carbon emissions of urban households. In terms of the variable parameters of economic urbanization, the highest coefficients of economic urbanization are found in the northwest and east regions of China, with higher variable parameters in the wider region from the northwest to the southwest, and the gradient of variable parameters becoming lower in the western and southwest regions. In terms of the variable parameters of lifestyle urbanization, the highest parameters are found in Xinjiang and Tibet in the west and Heilongjiang and other provinces and autonomous regions in the northeast, while the lowest parameters are found in the southeast. In terms of the variable parameters of urbanization of geographical landscapes, the variable parameters are highest in the southeast coastal region, middle in the vast central and western regions, lower in northern and northeastern China, and lowest in some regions of western China.

The results of the geographically weighted regression analysis show that urbanization factors have a contributing effect on some cities’ carbon emissions of urban households. So, as the level of urbanization increases, some cities’ carbon emissions of urban households in China increase instead. The empirical analysis in this paper reflects the characteristics of China’s urban household carbon emissions during the rapid urbanization phase. In particular, it is important to note that the impact of urbanization factors on urban household carbon emissions differs across different stages of urbanization. As the results of the geographically weighted regression analysis show, the effects of different urbanization factors on urban household carbon emissions show significant differences across different stages of urbanization development in northeastern, eastern, central, and western China.

## 6. Discussion and Conclusions

### 6.1. Discussion

#### 6.1.1. Discussion on the Application of the Composite Urbanization Score Measure

The main methods of measuring the comprehensive urbanization score are entropy, hierarchical analysis, factor analysis, weighted average, standardized score processing, and other analytical methods. Due to the differences in the geographical areas studied and the ease of obtaining indicator data, the selection of indicators and their focus also vary greatly, and the specific calculation methods used are also varied. This study uses the entropy method to measure the urbanization score of Chinese cities. The advantages of this method are that it can measure objective weights based on the differences in indicators, which is more objective than the subjective weights determined by the hierarchical analysis method; it retains the integrity of the indicator data compared to the factor analysis method, which loses information to a certain extent by reducing the dimensionality of variables. The urbanization score determined by the entropy method is not only able to analyze the overall urbanization score, but also the scores of secondary indicators of urbanization. This study analyses the scores of secondary indicators such as population urbanization, economic urbanization, lifestyle urbanization, and geographical landscape urbanization, to provide a relatively more comprehensive understanding of the quality of urbanization development in Chinese cities at the prefecture-level and above.

#### 6.1.2. Discussion on the Relationship between Urbanization and Carbon Emissions of Urban Households

In general agreement with Ou [4], we similarly argue that, during the rapid urbanization phase, China’s urbanization expansion was relatively crude during the study period, which is not conducive to carbon emission reduction, but at the same time, the mechanisms of urbanization factors acting on urban household emissions should also be viewed dialectically. On the one hand, China’s urbanization has obvious spatial differences, with significant differences in the gradients between the east, the center, and the west. China’s large urban agglomerations are the main areas of population migration, and rapid population growth will obviously put pressure on the carbon emissions of urban households in cities with population inflows. On the other hand, it is the driving force of the urbanization factor that has promoted a high degree of population agglomeration and improved the past state of fragmentation, which in turn has facilitated a reduction in carbon emissions for urban households in cities with population outflow.

### 6.2. Conclusions

The entropy method, Exploratory Spatial Data Analysis (ESDA), and geographically weighted regression analysis were used to construct a comprehensive index system for measuring urbanization level and to explore the spatial and temporal variation of carbon emissions of urban households in China and their urbanization factors, and the following main conclusions were drawn.

First, the overall carbon emissions of urban households in China showed a decreasing trend during the study period, but there were significant differences in the carbon emissions of urban households in the four major regions. In terms of spatial patterns in the east and west, urban household carbon emissions are higher in the eastern region than in the central and western regions. In terms of North-South spatial patterns, household carbon emissions remain high in China’s livable regions, especially in key cities.

Second, in terms of the spatial and temporal characteristics of urban household carbon emissions, the urban “head effect” of urban household carbon emissions is obvious, that’s urban household carbon emissions in key cities are much higher than those in most cities. In terms of local spatial autocorrelation analysis, economically developed cities and livable areas in China are the areas with a high concentration of urban household carbon emissions.

Third, the relationship between urban household carbon emissions and urbanization shows that the four subsystems of urbanization are positively correlated with urban household carbon emissions in the same direction. Lifestyle urbanization is the most strongly correlated with urban household carbon emissions, followed by population urbanization, and the least strongly correlated with landscape urbanization.

Fourth, the results of the geographically weighted regression analysis show that urbanization factors have a contributory effect on some cities’ carbon emissions of urban households, but there are significant regional differences in the impact of urbanization factors on urban household carbon emissions in the eastern, central and western regions of China, as they are at different stages of rapid urbanization development.

## 7. Suggestions

An in-depth understanding of the relationship between urbanization and urban household carbon emissions in China must be analyzed in the context of China’s stage of urbanization development. Rapid urbanization has, on the one hand, energized China’s economic growth; on the other hand, it has created problems such as carbon emissions. Due to the different stages of urbanization development in the major regions of China, the development responses adopted vary.

First, the spatial governance of towns and cities in eastern China should be strengthened. Strengthen the top-level design of urban spatial governance in the eastern region and promote the synergistic development of “production, living and ecological” spaces [75]. Establish a collaborative mechanism for multi-sectoral cooperation in governance, establish a multi-dimensional collaborative spatial governance mechanism, and encourage residents and third-party subjects to participate in urban spatial governance.

Second, optimize the spatial layout of towns and cities in central China. Establish an institutional mechanism for the integration of multiple regulations, determine arable land and permanent basic farmland, ecological protection red lines and reasonable development boundaries for towns and cities, and optimize the spatial layout of towns and cities. We will follow the laws of urbanization, promote the coordinated development of large, medium, and small cities, improve the mechanism for joint construction of cities and towns, create a one-hour commuting circle, and promote the optimal development of large cities in central China.

Third, promote green and low-carbon development in western China. Green and low-carbon development is the fundamental way to solve the problem of carbon emissions from urban households. By integrating theoretical resources, building a value system, and finding a way to inject green development momentum (innovation-driven green transformation) → quality maintenance (ecological coordination and governance) → fair realization (open cooperation and sharing for all), a green low-carbon development path with Chinese characteristics has been constructed [76]. Actively promote Shanghai’s experience and speed up the separation of domestic waste in the western region. Promote green travel, reduce private car travel, and strengthen the construction of green families and green communities [77].

## Figures and Tables

**Table 1:** Carbon emissions from urban households (Unit: 10^4^ tons).

	Carbon Emission	Total Emissions		Average Emissions	
Region		2005	2015	2005	2015
Northeast Region		1553.93	1015.09	45.70	29.86
Eastern Region		5551.95	3543.73	63.82	40.73
Central Region		2590.71	2324.23	32.38	29.05
Western Region		2953.35	2983.51	34.75	35.10

**Table 2:** Classification of high-low carbon emission concentrations of urban households.

Year	2005	2015
High-High Cluster (HH)	Harbin, Changchun, Beijing, Tianjin, Shijiazhuang, Tangshan, Langfang, Baoding, Zhangjiakou, Cangzhou, Xingtai, Handan, Jinan, Weifang, Qingdao, Yantai, Shanghai, Foshan, Datong, Taiyuan, Hohhot, Ulanqab	Beijing, Tianjin, Tangshan, Baoding, Shijiazhuang, Handan, Shanghai, Datong, Taiyuan, Xi’an, Chengdu, Chongqing, Deyang
High-Low Cluster(HL)	Xining, Chengdu, Chongqing, Wuhan	
Low-High Cluster(HL)	Xilingole	

**Table 3:** Comprehensive evaluation index system of urbanization level.

Secondary Indicators	Tertiary Indicators	Unit
Population urbanization	Urban population as a proportion of the total population	%
	Population size in urban areas	10,000 people
	Population density in urban areas	people/km^2^
	Share of employees in the secondary sector	%
	Share of tertiary sector employees	%
Economic urbanization	GDP per capita	¥
	GDP growth rate	%
	Share of secondary sector in GDP	%
	Tertiary sector as a share of GDP	%
	Total industrial output value per capita above scale	¥
Lifestyle Urbanization	Number of hospital beds per 10,000 population	pieces
	Number of university students per 10,000 population	people
	The number of people employed in urban units at the end of the period for 10,000 people	people
	Post and telecommunications operations per capita	¥
	Retail sales of social consumer goods per capita	¥
Geographical landscape urbanization	Urban built-up land as a proportion of urban area	%
	Urban road area per capita	m^2^
	Built-up area per capita	km^2^
	Fixed asset investment per capita	¥
	Greenery coverage in built-up areas	%

**Table 4:** Overall score of urbanization.

Overall Score	Eastern Region	Central Region	Western Region	Northeast Region	Sum
Top25%	34 (11.89%)	16 (5.59%)	16 (5.59%)	6 (2.10%)	72 (25.17%)
Middle50%	43 (15.03%)	48 (16.78%)	29 (10.14%)	22 (7.69%)	142 (49.65%)
Bottom25%	10 (3.50%)	16 (5.59%)	40 (13.99%)	6 (2.10%)	72 (25.17%)
sum	87 (30.42%)	80 (27.97%)	85 (29.72%)	34 (11.89%)	286 (100%)

**Table 5:** Secondary indicators score of urbanization.

Secondary Indicators	Ranking	Eastern Region	Central Region	Western Region	Northeast Region
Population urbanization	Top25%	21 (7.34%)	18 (6.29%)	17 (5.94%)	16 (5.59%)
	Middle50%	56 (19.58%)	43 (15.03%)	27 (9.44%)	16 (5.59%)
	Bottom25%	10 (3.50%)	19 (6.64%)	41 (14.34%)	2 (0.70%)
Economic urbanization	Top25%	40 (13.99%)	14 (4.90%)	12 (4.20%)	6 (2.10%)
	Middle50%	41 (14.34%)	50 (17.48%)	36 (12.59%)	15 (5.24%)
	Bottom25%	6 (2.10%)	16 (5.59%)	37 (12.94%)	13 (4.55%)
Lifestyle Urbanization	Top25%	35 (12.24%)	13 (4.55%)	17 (5.94%)	7 (2.45%)
	Middle50%	42 (14.69%)	52 (18.18%)	33 (11.54%)	15 (5.24%)
	Bottom25%	10 (3.50%)	15 (5.24%)	35 (12.24%)	12 (4.20%)
Landscape urbanization	Top25%	33 (11.54%)	22 (7.69%)	13 (4.55%)	4 (1.40%)
	Middle50%	45 (15.73%)	41 (14.34%)	39 (13.64%)	17 (5.94%)
	Bottom25%	9 (3.15%)	17 (5.94%)	33 (11.54%)	13 (4.55%)

**Table 6:** Pearson correlation analysis between urban household carbon emissions and urbanization factors.

Secondary Indicators	Tertiary Indicators	Correlation Coefficients	Level of Significance
Population urbanization	Urban population as a proportion of total population	0.248	*p* < 0.01
	Population size in urban areas	0.746	*p* < 0.01
	Population density in urban areas	0.062	Insignificant
	Share of employees in the secondary sector	−0.065	Insignificant
	Share of tertiary sector employees	0.075	Insignificant
Economic urbanization	GDP per capita	0.177	*p* < 0.01
	GDP growth rate	0.061	Insignificant
	Share of secondary sector in GDP	−0.140	*p* < 0.05
	Tertiary sector as a share of GDP	0.245	*p* < 0.01
	Total industrial output value per capita above scale	−0.049	Insignificant
Lifestyle Urbanization	Number of hospital beds per 10,000 population	−0.116	Insignificant
	Number of university students per 10,000 population	0.321	*p* < 0.01
	The number of people employed in urban units at the end of the period for 10,000 people	0.335	*p* < 0.01
	Post and telecommunications operations per capita	−0.034	Insignificant
	Retail sales of social consumer goods per capita	0.260	*p* < 0.01
Geographical landscape urbanization	Urban built-up land as a proportion of urban area	0.170	*p* < 0.01
	Urban road area per capita	−0.019	Insignificant
	Built-up area per capita	0.066	Insignificant
	Fixed asset investment per capita	0.094	Insignificant
	Greenery coverage in built-up areas	0.039	Insignificant

## Data Availability

All data are available in the public domain.
